# Fast Recruitment of Recurrent Inhibition in the Cat Visual Cortex

**DOI:** 10.1371/journal.pone.0040601

**Published:** 2012-07-25

**Authors:** Ora Ohana, Hanspeter Portner, Kevan A. C. Martin

**Affiliations:** 1 Institute for Molecular and Cellular Cognition, Center for Molecular Neurobiology, ZMNH, University Medical Center Hamburg-Eppendorf, Hamburg, Germany; 2 Institute of Terrestrial Ecosystems, ETH Zurich, Zurich, Switzerland; 3 Institute of Neuroinformatics, University of Zurich/ETH Zurich, Zurich, Switzerland; University of Southern California, United States of America

## Abstract

Neurons of the same column in L4 of the cat visual cortex are likely to share the same sensory input from the same region of the visual field. Using visually-guided patch clamp recordings we investigated the biophysical properties of the synapses of neighboring layer 4 neurons. We recorded synaptic connections between all types of excitatory and inhibitory neurons in L4. The E–E, E–I, and I–E connections had moderate CVs and failure rates. However, E–I connections had larger amplitudes, faster rise-times, and shorter latencies. Identification of the sites of putative synaptic contacts together with compartmental simulations on 3D reconstructed cells, suggested that E–I synapses tended to be located on proximal dendritic branches, which would explain their larger EPSP amplitudes and faster kinetics. Excitatory and inhibitory synapses were located at the same distance on distal dendrites of excitatory neurons. We hypothesize that this co-localization and the fast recruitment of local inhibition provides an efficient means of modulating excitation in a precisely timed way.

## Introduction

The aim of this study was to describe the statistical and kinetic properties of synaptic connections in layer 4 of the cat’s primary visual cortex. The reason for this interest is that while the role of thalamic afferents in producing simple receptive fields is well-studied (reviewed by [1]–[3]), far less is known on the synaptic interactions of the neurons within layer 4. Neurons comprising the layer 4 network are excitatory spiny stellate and star pyramidal neurons, and the inhibitory neurons, like basket cells. Thalamic axons form synapses with both excitatory and inhibitory layer 4 neurons, but provide only about 5% of their synapses [4]. The majority of synapses on layer 4 neurons are provided by spiny stellate cells and layer 6 pyramidal cells [5].

Previous studies of the physiological properties of intracortical synapses in layer 4 of the cat visual cortex revealed a network of moderately strong and variable excitatory synapses but highly reliable inhibitory synapses [6]–[13]. These findings strengthen the hypothesis that the recurrent excitatory connections act to amplify the transient thalamic input, while the recurrent inhibition serves to balance the excitation and prevent runaway excitation [14], [15].

Temporal factors would seem important in this interaction between excitation and inhibition, but little is known about the kinetic properties of EPSPs and IPSPs generated by neurons in cat layer 4. This is in part due to the technical limitations of previous studies, but also because precise synaptic timing on the millisecond time scale has not previously been considered to be a major factor in visual information processing. However, recent theoretical studies have indicated that precise timing of thalamic inputs may well be an essential factor in driving cat simple cells [16], [17]. Precise timing of the feedforward excitatory and inhibitory inputs were also shown in rodent somatosensory and auditory cortices [18], [19], so it seemed likely to us that synaptic dynamics may also play an important role in modulating the response of layer 4 cells. To define some key aspects of the synapses formed between cat layer 4 neurons, we employed visually-targeted dual whole cell patch clamp recordings from neighboring L4 neurons in acute slices of cat V1 to measure the kinetics and latencies of their PSPs. Our data show that excitatory synapses onto inhibitory L4 cells have the largest amplitudes, fastest rise and decay kinetics and are evoked with the shortest latencies. Based on compartmental modeling of the reconstructed neurons and on putative synaptic locations, we suggest that the uniquely fast recruitment of E–I synapses results from their proximal location on the inhibitory dendrites. We show that this mechanism provides an additional means of modulating the activity of recurrent circuits.

## Methods

### Ethics Statement

All animal experiments were approved by the Kantonal Veterinaeramt of Zurich and performed under License Nr. 25/2001 and 50/2003 to K.A.C.M.

### Slice Preparation

Performing visually-targeted patch clamp recordings requires that the slices be particularly healthy and that their surfaces contain numerous viable cells. Since this mode of recording was not previously performed in adult cat slices, procedures were first optimized to meet these requirements and are subsequently described in detail. Recordings were made in slices of visual cortex taken in a terminal procedure from 2–24 months old male and female cats that had been used in in-vivo experiments. Anesthesia was induced by subcutaneous injection of xylazine (Rompun, Bayer, 0.5 mg/kg) and ketamine (Narketan 10 mg/kg, or Vetoquinol, 10 mg/kg) and maintained with intravenous injections of alphaxalone/alphadolone (“Saffan”, Schering-Plough Animal Health) while the animal was placed in a streotaxic apparatus. The visual cortex was accessed by craniotomy and a block of cortex was excised and put immediately into iced slicing-ACSF. The block was trimmed such that it contained the medial bank, areas 17 and 18, and glued with cyanoacrylate onto the surface of a rotating metal plate. The plate was placed in the slicing-chamber and rotated such that the top of the lateral gyrus was always perpendicular to the blade. The chamber was constantly filled with freshly iced slicing-ACSF and continuously oxygenated (95% O_2_– 5% CO_2_). Coronal slices (300 µm) were cut on a vibratome (Sigmann Elektroniks, Germany) at a speed of 0.7 mm/s and at lateral vibration amplitude of 1.4 mm and 40 Hz vibration frequency. Slices were maintained in a submersion chamber containing oxygenated recording-ACSF for 45 min at 37° and subsequently at room temperature (21°–24°). Slices obtained from cats older than 6 months were maintained in slicing-ACSF to improve neuronal viability. ACSF contained, in mM: 125 NaCl, 2.5 KCl, 25 NaHCO_3_, 1.25 NaH_2_PO_4_, 1 MgCl_2_, 2 CaCl_2_, 10 Glucose. Slicing-ACSF contained in addition: 3 Myo-Inositol, 2 Na-Pyruvate, 0.4 Ascorbic acid and a total of 25 mM Glucose. Slices could be maintained up to 24 Hr after slicing, however, stable recordings were typically obtained from slices <18 Hr.

### Electrophysiology

Slices were placed in a submersion chamber and continuously perfused with warmed oxygenated ACSF at a rate of 2–3 ml/min, temperature at the slice was 34–36°. Neurons were visualized with an IR-DIC equipped microscope at ×60 magnification. Somatic whole-cell patch-clamp recordings were made in current-clamp mode (Multiclamp-700a amplifier, Axon Instruments, Foster City, CA) from visually identified L4 neurons in area 17 of the visual cortex. Data acquisition was done on-line through an AD converter (Digidata 1322, Axon Instruments, Foster City, CA) at sampling rates of 10 kHz and filtered at 3 kHz. Access resistance was continuously monitored and the bridge potential was compensated. Patch pipettes were pulled from borosilicate capillaries (2 mm outer diameter, 0.5 mm wall thickness, Hilgenberg, Germany). Typically, pipettes had tip-diameters of 1.5–2.5 µm and a 4–8 MΩ resistance when filled with recording pipette solution, under these conditions the access resistance was 6–25 MΩ. EPSPs were evoked by injecting either single or trains of current pulses (4–9 pulses, each 2–4 ms, 0.6–1.4 nA) into the presynaptic neuron at inter-train frequencies of 1–100 Hz, trains were repeated at a rate of 0.1–0.125 Hz.

Pipette solution contained, in mM: 135 K-Gluconate, 4 Mg-ATP, 5 Na2-Phosphocreatin, 0.3 GTP, 10 Hepes, KCl was added in concentrations between 4–20 mM, pH was adjusted to 7.2 and osmolarity to 280–290 mOsmolar. Biocytin (0.25–0.5%) was added to the pipette solution prior to recording.

Neurons in layer 4, particularly spiny stellates, had shown high sensitivity to the recording conditions. Pipette solutions containing low [Cl^−^] were used in an attempt to facilitate the detection of inhibitory GABA_A_ synaptic connections. However, while IPSPs could be well detected and recorded, the measured reversal potential of the IPSP did not depend on the pipette [Cl^−^]. On the other hand, low [Cl^−^] caused rapid swelling of layer 4 somata and dendrites and a partial loss of dendritic spines. Pyramidal neurons in layers 3 and 5 did not show similar structural damage or swelling. Using smaller tip pipettes with resistance of 6–8 MΩ reduced the damage to layer 4 neurons and recording with high [Cl^−^] (20 mM) prevented the damage. In addition, neurons in slices obtained from cats older than 3 months were more susceptible to structural damage, swelling, and depolarization of membrane potential than neurons from younger cats.

### Histology

Immediately following recordings, slices were fixed in 4% paraformaldehyde in 0.1 M phosphate buffer (PB), pH 7.4 and left overnight. Slices were then washed 3 times in PB, incubated for 5 min in 1% H_2_O_2_ in PB to quench endogenous peroxidase and subsequently washed in PB (×4, 10 min each). Membranes were permeabilised by incubation with 1% Triton ×100 for 2 Hr (4 Hr for slices obtained from cats >6 months). Slices were then reacted in ABC kit (Vectostain elite, Reactolab) containing 0.5% Triton ×100 for 3 hr in room temperature. Following a series of washes in PB, slices were incubated in 0.05% diaminobenzodine in PB containing 0.1% H_2_O_2_ for 5–10 min until cells became sufficiently dark. Staining was terminated by washes in PB. Slices were placed on a microscope slide and embedded in Moviol (Fluka) after excess liquid was removed. If not otherwise stated, chemicals were obtained from Sigma-Aldrich.

The 3-D neuronal morphology was reconstructed in the light microscope (LM) with a computerized system (Neurolucida, MicroBrighField) equipped with a ×60 or ×100 oil objective and analyzed with Neurolucida Explorer (version 4.50.2). The morphological criteria for determination of a contact were the existence of a varicosity or boutons, and no discernible gap between the preysnaptic bouton and the postsynaptic dendrite.

### Analysis

Amplitudes of unitary PSPs (EPSPs or IPSPs) were calculated as the mean of 21 points around the peak amplitude within a time window of 5–10 ms after the presynaptic AP. CV was calculated as follows: Failures of the unitary PSPs were manually detected by comparing single traces with the averaged PSP template, and are expressed as percentage of all monitored traces. The release probability (*p*) was calculated from the following equation [20]:
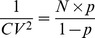



The number of release sites N was assumed to be the same as the number of contacts determined from the 3-D reconstruction, in few cases where N was not morphologically determined, the averaged N of the connection type was used.

For analysis of the PSP kinetics, 20–50 traces were selected in which a PSP was evoked. These traces were aligned to the peak of the presynaptic APs, their baseline potential subtracted and averaged. The EPSP latency was calculated as the interval between the time of the preysnaptic AP peak and the time at which the EPSP rose to 10% of its maximal amplitude. The rise time was calculated as the interval between 10–90% rise of the EPSP amplitude and the half width was calculated as the time interval between the rise and decay of the EPSP at the 50% peak amplitude of the EPSP. The analysis was performed on the aligned averaged trace only.

All means are represented ± s.d. unless otherwise stated. Statistical significance was tested with non-parametric test due to small sample size. The Mann-Whitney U test was used for comparison of 2 groups and the Kruskal-Wallis test for 3 groups.

### Compartmental Simulation

All simulations were carried out using the NEURON simulation environment Version 5.9 [21] either on a Pentium PC running Linux (the evolutionary algorithms) or on a PC running Windows XP. The integration time step used was 25 µs. Four inhibitory (basket) and six excitatory (5 stellate and 1 star pyramid) cells were used for the compartmental modeling. The passive properties were measured in these neurons and they could be fully reconstructed. The Neurolucida reconstruction files were converted to NEURON format using NeuroConvert43 (Arnd Roth & Klaus Bauer MPI Medizinische Forschung, Heidelberg). Reconstructed spines were deleted from the Neurolucida files prior to conversion. Neurons were segmented into compartments whose length was proportional to the square root of the local diameter.

To determine their passive parameters, simulated voltage responses to somatic current pulses were directly fitted to the experimentally measured voltage responses in the same cell. An evolutionary algorithm written in (C++) was applied to explore the parameter space and was instructed by a least-square error function of the fits to find the best matching values of the specific membrane resistance (Rm), specific membrane capacitance (Cm) and the axial resistivity (Ri). The population size was 60 and the algorithm was repeated over 10 generations.

Spines were globally incorporated into the dendrites by locally multiplying Cm and 1/Rm in by a factor of 1.8. This factor was calculated as follows: F = (Nspines* DF * ASpine +ADendrites)/ADendrites. Where NSpines is the total number of reconstructed spines (6755 from all spiny stellate cells, n = 6), DF is the ratio between the spine density reported in EM studies (0.35/µm) and the one observed in our LM reconstructions (0.19/µm), ASpine is the spine area calculated in Neurolucida Explorer (4.25 µm^2^, approximated by a Frustum) and ADendrites is the total dendritic area of the reconstructed spiny stellate cells (95,586 µm^2^). The excitatory neurons were simulated twice: once without spines and once with the scaling factor. The evolutionary algorithm fit yielded the following average uniform passive parameters for 4 smooth cells: Cm = 1.3±0.44 µF/cm^2^ (range: 0.76–1.84); Rm = 3938±2134 Ωcm^2^ (range: 2006–6987) and Ri = 118±55 Ωcm (range: 60–173). The fitted properties of the excitatory neurons (n = 6) without spines were: Cm = 1.2±0.58 µF/cm^2^ (range: 0.4–1.82); Rm = 11124±6755 Ωcm^2^ (range: 5270–22000) and Ri = 134±46 Ωcm (range: 72–181). With spine scaling factor of 1.8 the fitted properties were somewhat different: Cm = 0.85±0.34 µF/cm^2^ (range: 0.35–1.23); Rm = 16107±6921 Ωcm^2^ (range: 9723–27516) and Ri = 128±57 Ωcm (range: 67–219). In all simulations the reversal potential (Erev) was set at −64 mV and the temperature at 34°C for both cell types. It should be noted that these parameters are not truly “passive”, since the experiments were performed without any blockers of ionic or synaptic conductances, but rather represent the effective membrane conductances active at voltages near the resting membrane potential.

Simulations of the synaptic responses were performed on 6 excitatory and 4 inhibitory neurons each assigned its own fitted passive parameters. The data for the latencies, attenuation and rise times were obtained by successively attaching the same artificial spine segment to each dendritic compartment in the model neuron and stimulating its inserted conductances. The spine’s length and diameter were 1 µm and 0.35 µm, respectively. The spine had the same passive properties as the rest of the cell and included an AMPA and an NMDA current. The AMPA synapse was modeled as a sum of 2 exponentials according to NEURON’s built in Exp2syn point process with the following parameters: gmax = 0.0038 µS, τ1 = 0.23 ms and τ2 = 1 ms for the fast synapse (used for the inhibitory neurons only) and τ2 = 2.3 ms for the slow synapse. The NMDA current was simulated by a series of functions written by Arnd Roth (1994) taking into account Mg^2+^ block and partial calcium permeability with the following parameters: gmax = 0.0005 µΩ/cm2, τ1 = 2.5 ms and τ2 = 30 ms. The simulated time constants were based on voltage clamp measurements of isolated AMPA and NMDA EPSCs recorded from single-axon synapses between cortical L5 neurons (Ohana; unpublished results). The synaptic conductance was deliberately set at a high value to ensure that the EPSP from all dendritic compartments was visible at the soma. All results shown in the figures were obtained using this standard model. The latency was defined as the time from stimulus onset in the spine to 10% of the EPSP peak amplitude in the soma, a fixed value of 0.3 ms was added to the simulated latency to account for axonal propagation and transmitter release time. The attenuation was calculated as the ratio of the peak EPSP in the soma over its amplitude in the spine. The rise time was calculated from the EPSP trace in the soma, as the time difference between 10% and 90% peak amplitude.

### Network Simulations

A network of 320 excitatory and 80 inhibitory current-based leaky integrate-and-fire (LIF) neurons (modified from NEURON’s IntFire4 mod file [21]–[23] was implemented in NEURON version 5.6 (please refer to Appendix S1 for details). Thalamic input was modeled with a layer of 400 “thalamic cells” (NEURONs NetStim object) which are essentially stimulus generators providing synaptic input once activated. Cells in the network were connected randomly with connection probabilities chosen such that every L4 excitatory neuron received: 45 thalamic (18%), 160 recurrent excitatory (64%) and 45 inhibitory (18%) synaptic inputs. Inhibitory neurons received: 40 thalamic (18%), 140 recurrent excitatory (64%) and 40 inhibitory (18%) synaptic inputs. The proportions of excitatory and inhibitory neurons in the network reflect published anatomical values but the relative proportions of synaptic sources reflect a functional rather than an anatomical structure. All LIF neurons had a membrane time constant of 20 ms and received inhibitory, excitatory and thalamic input with various kinetics (for theoretical explanation see [23,23]. All synapses were modeled with a double-exponential function. Thalamic input synapses (thalamic-E, thalamic-I) were modeled with the following parameters: τ1 = 0.23 ms and τ2 = 2.3 ms and a latency of 0 ms. E–E and E–I synapses were modeled with τ1 = 0.23 ms and τ2 = 2.3 ms and a latency of 1.4 ms. Inhibitory synapses (I–I and I–E) were modeled with τ1 = 0.23 ms and τ2 = 3 ms and a latency of 1.4 ms. The fast E–I synapse was modeled with a τ1 = 0.23 ms, τ2 = 1 ms and a latency of 0.5 ms. Synaptic weights (W) for each individual connection were randomly assigned from a Gaussian distribution with a median of W and a variance of σ. The random distribution was generated by an ACG algorithm (intrinsic to NEURON) with a fixed seed, such that in every run of the simulations the same random variables were selected. This was done to ensure that only the synaptic kinetics vary between the different simulation runs. The values of the weight were as follows: T-E W = 0.004375 and σ = 0.0021875, T-I W = 0.0055 and σ = 0.0021875, E–E W = 0.0055 and σ = 0.0005, I–E, and I–I had W = −0.0055 and σ = 0.0005 E–I = 0.011 and σ = 0.0011. Input stimulus. Thalamic input synapses were activated by 250 ms long trains of APs whose times were generated using a Poisson process with underlying average rate *F_in_*. The latter was calculated according to the equation adapted from [24] as follows:
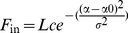
Where α is the tuning factor and α0 represents the preferred tuning factor (range 0,1), σ is the bandwidth of the tuning function, L is the maximal frequency and set to 80 Hz and C is the intensity factor (range 0,1), was set to 0.4. The L4 model neuron response is measured as the mean firing rate during the 250 ms input stimulus.

### Synaptic Conductance Calculations

Because we measured the synaptic potential rather than the currents and because most synapses were located distal to the soma, we could not provide direct estimates of the synaptic conductance. We calculated the synaptic conductance from Ohm’s law, as follows:
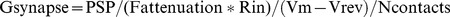



PSP was the averaged PSP amplitude, Fattenuation was the simulated passive attenuation factor of the PSP from the “overlap” compartments to the soma. For the E–E and I–E conductances Fattenuation was taken from the simulations of the excitatory synapses on the excitatory neurons and was 0.046 on average. For the E–I conductance, Fattenuation was taken from the simulations of the excitatory synapses on the inhibitory neurons and was 0.39 on average. Rin was the averaged value given in Table 1. Vm was the membrane potential at which the PSPs were recorded and that was on average −66 mV, −64 mV and −51 mV, for the E–E, E–I and I–E synapses, respectively. Vrev was the reversal potential of the excitatory synapses was assumed to be 0 mV while Vrev of the inhibitory synapses was set to −67 mV, as we measured (see Results). Ncontacts was taken from the morphological reconstructions and was 3, 2 and 5 for the E–E, E–I and I–E, respectively.

**Table 1:** Reliability of EPSPs and IPSPs.

	Amplitude (mV)	CV	Failures (%)	Distance (µm)
**E–E**	0.38±0.35 (0.073–1.33) [n = 11]	0.424±0.155 (0.293–0.753) [n = 7]	20±25 (5–74) [n = 7]	47±44 (5–116) [n = 10]
**E–I**	1.54±1.55 (0.224–4.2) [n = 8]	0.332±0.154 (0.17–0.53) [n = 7]	17±28 (0–68) [n = 7]	40±40 (16–137) [n = 8]
**I–E**	0.598±0.4 (0.18–1.44) [n = 9]	0.295±0.12 (0.129–0.432) [n = 9]	6.4±11 (0–34) [n = 9]	27±15 (3–51) [n = 8]
**I–I**	0.78 [n = 1]	0.369 [n = 1]	5 [n = 1]	21 [n = 1]

Amp, amplitude; CV, coefficient of veriation; Distance, between pre- and post somata as measured from reconstruction. Means are presented ± s.d. Ranges from min to max values are written in parentheses and number of observations in square brackets.

## Results

### Layer 4 Synaptic Connections Recorded with Dual Patch-clamp

**Figure 1:**
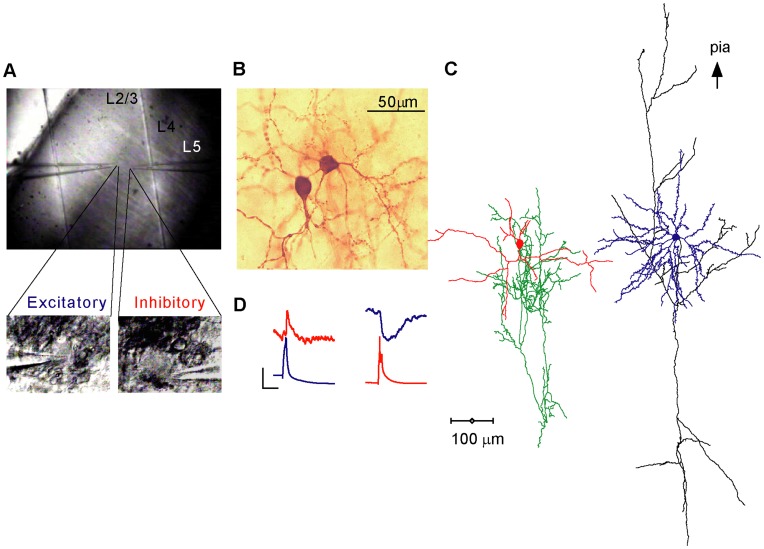
Paired recordings from visually identified L4 neurons. **A**. An IR-DIC image of a coronal slice from area 17 of the cat cortex. Layer 4 was identified under low magnification (10×) as a dark stripe extending over the middle third portion of the cortex. Lower panel: at a higher magnification (60×) an excitatory neuron (left) and an inhibitory neuron (right) were recorded simultaneously with patch clamp pipettes. **B, C**. Biocytin stain of the same cell pair and its 3-D reconstruction, identifying them as a spiny stellate cell and a smooth basket cell. The cells are presented separately for clarity. **D**. The cell pair was reciprocally connected as is evident by the EPSP (red) and IPSP (blue) evoked in the basket and spiny stellate neurons, respectively. The presynaptic APs in the basket and stellate cells are colored blue and red respectively. Vertical scale bar is 0.4 mV for the EPSP/IPSP and 50 mV for the APs and horizontal scale bar is 20 ms.

An example of a dual patch clamp experiment is illustrated in Fig. 1. The low magnification IR-DIC image of the coronal slice (×10, Fig. 1A, upper panel) shows layer 4, which is visible as a dark band at 600–1000 µm below the pial surface. Specific neurons were selected for recording based on morphological cues that were observed at higher magnification (×60, Fig. 1A, lower panels). In this experiment a presumed spiny stellate neuron was selected, based on its relatively small and quadratic appearance, while the presumed smooth neuron was selected based on its round shape and the stubby short dendrite emerging from the soma (below the pipette tip). Staining for biocytin and 3-D reconstruction (Fig. 1B, C) of the recorded neurons, confirmed their presumed identity and provided the detailed morphological information for simulations and for identification of putative synaptic contact sites. Most recorded neurons were successfully filled with biocytin, so that in all but 2 cases the identities of the neurons in the pair were confirmed morphologically. Synaptic connections were tested by alternately driving one of the two neurons to produce single action potentials, or more commonly, trains of action potentials (APs). Fig. 1D, shows the averaged EPSP evoked in the smooth basket neuron by the AP discharged in the spiny stellate neuron, and the reciprocal IPSP evoked in the spiny stellate neuron in response to an AP in the basket neuron.

In approximately 70 slices obtained from 20 cats, we tested 464 neuron pairs of which 25 were synaptically connected (6.3%, bi-directionally tested). There were 10 pairs between two excitatory neurons, 14 between an excitatory and an inhibitory neuron and a single inhibitory-inhibitory pair. Four of the pairs (14%) were bi-directionally connected (1 E–E and 3 E–I). Thus out of 25 pairs, a total of 29 connections were measured, of which 11 were E–E, 8 E–I, 9 I–E and 1 I–I.

### Morphology of Pairs

Morphological reconstructions provide detailed information on the axonal and dendritic structure of the recorded neurons. This information can be used to define microcircuits within the cortex and for understanding the properties of the recorded synapses. Since, such a correlative study has not previously been made for the excitatory network of cat layer 4, we routinely stained all recorded neuron pairs and reconstructed in 3-D the dendritic and axonal arbors of most.

**Figure 2:**
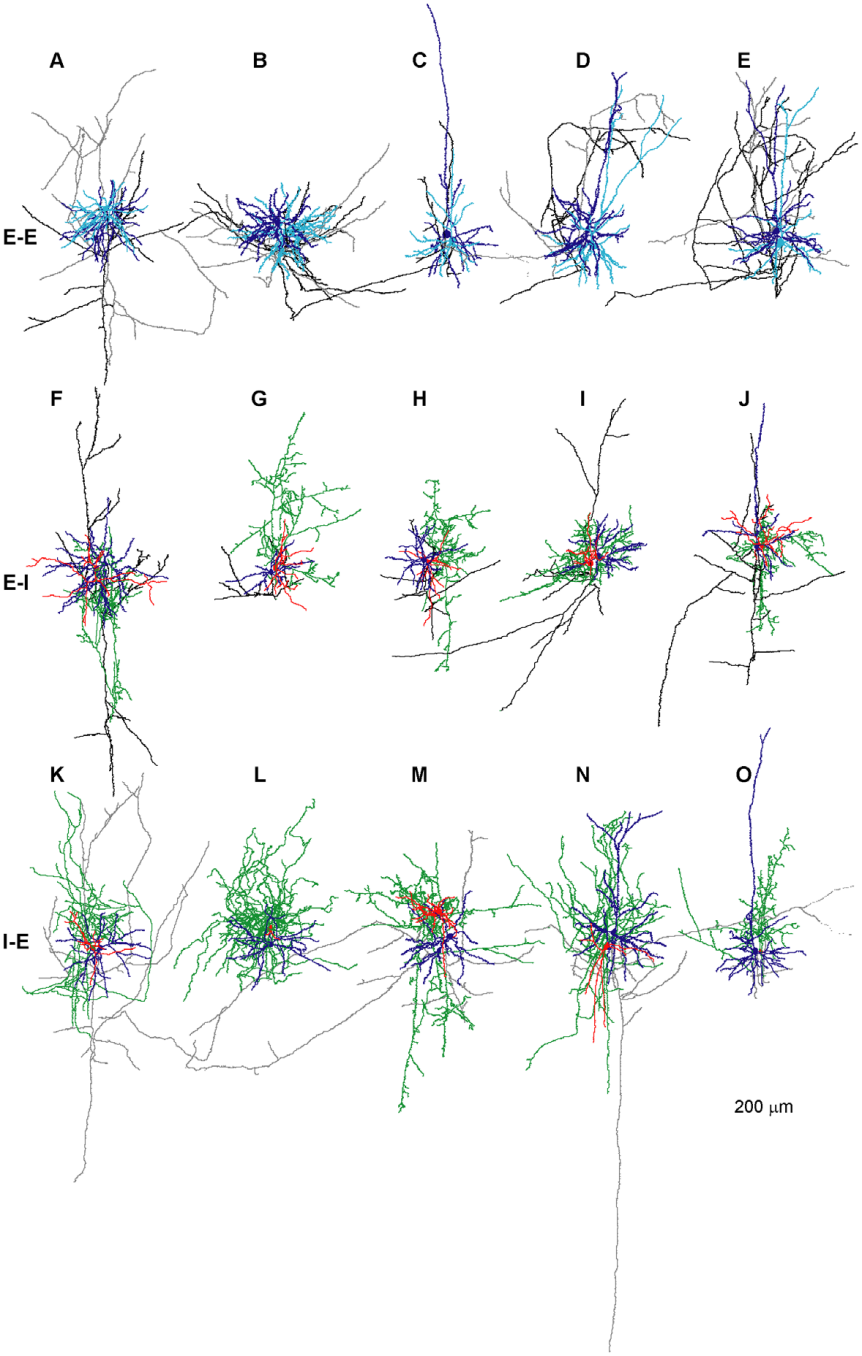
Reconstructed 3D morphology of layer 4 pairs. **A–E**. illustrating 5 E–E pairs. A, B pairs of spiny stellate neurons. C, D, E pairs of star pyramidal neurons. Preynaptic excitatory cells are cyan colored and their axons colored black. Postsynaptic excitatory cells are colored blue and their axons grey. **F–J** illustrates 5 pairs of excitatory and inhibitory neurons connected via an E–I synapse. In F–I the presynaptic neuron was a spiny stellate, in J a star pyramid. The postsynaptic neurons in F–J are all basket cells. Presynaptic excitatory cells are colored blue and their axons black, postsynaptic basket cells are colored red and their axons green. **K–O** Pairs of basket cells contacting excitatory spiny stellate (K–M) or star pyramidal (N–O) cells. Note the dense local ramifications of the basket cells’ axons. Presynaptic basket cells are red and their axons green, postsynaptic stellate and star pyramidal cells are blue and their axons grey. For presentation purposes all dendrites and axons were drawn with the same line thickness. Cell pairs **F** and **I** were reciprocally connected.

The major neuron types in layer 4 are star pyramids, spiny stellates, and smooth multipolar cells. Star pyramids and spiny stellates, which are excitatory, were easily identified by their stereotypical dendritic morphology [25], [26], most notably the existence of numerous dendritic spines, radially emerging basal dendrites and an apical dendrite (star-pyramids). Smooth inhibitory cells are more variable in form and size and were classified by a combination of dendritic and axonal attributes. We identified inhibitory cells by their smooth, beaded dendrites and by their densely ramifying, curved axon collaterals that were richly studded with large synaptic boutons [26]–[28]. Excitatory connections were found exclusively between pairs of spiny stellate (5/9 pairs) or between pyramidal neurons (4/9). Six excitatory neurons presynaptic to a smooth inhibitory cell were spiny stellates and two were star pyramids. Smooth inhibitory cells targeted 3 star pyramids and 6 spiny stellate cells. In one pair (Fig. 2N) the presynaptic neuron was a layer 4 basket cells whereas the postsynaptic neuron was a layer 3-like pyramidal cell at the border of layer 4 and 3. Thus our sample of close-range layer 4 synapses includes connections between similar excitatory neurons and connections between excitatory and inhibitory neurons, involving all cell types.

The morphology of 15 pairs (E–E, E–I, I–E, 5 of each) was sufficiently well-preserved to allow full reconstruction of 20 excitatory and 10 inhibitory neurons. Excitatory cells were classified as star pyramids (n = 9) and spiny stellate neurons (n = 11). Star pyramids had larger somata (mean diameter 20.2±4.2 µm, n = 9 vs. 16.1±1.8 µm, n = 11, P = 0.012 Mann-Whitney U-Test) but fewer primary dendrites (5.4±1.7 n = 9 vs. 7.7±1.8, n = 11, P = 0.009 Mann-Whitney U-Test) and shorter total dendritic length than the spiny stellates (2190±754 µm, n = 9 vs. 3218±1155 µm, n = 11, P = 0.04 Mann-Whitney U-Test). However, the basal dendritic span and total spine numbers were similar (147±20 µm vs. 147±28 µm, P = 0.73 and 854±360 vs. 614±290 spines, P = 0.12 Mann-Whitney U-Test, n = 9 and n = 11 for star pyramids and stellate cells, respectively). These data suggest that excitatory synapses onto stellate and star pyramidal cells of layer 4 are distributed similarly. Axons of both excitatory types had similar length (3618±2033 µm, n = 8 and 4075±1716 µm, n = 10, P = 0.48 Mann-Whitney U-Test, strongly severed axons (<850 µm in length) were omitted from the analysis).

All 10 reconstructed inhibitory neurons were classified as basket cells owing to their radially extending dendrites (Fig. 2F–J, 2K, 2L–O), dense clusters of large boutons and curving axons, which occasionally formed multiple contacts around somata. Eight basket cells had small-to-medium sized somata (averaged diameter 15.7±2.6 µm, n = 8), but two (Fig. 2M, 2N) had larger somata (mean diameter 24 and 22 µm, respectively) and a wider axonal span. These two were medium-sized basket cells. All inhibitory neurons had narrow action potentials (AP half width as measured from the AP threshold was on average 0.4±0.13 ms, range 0.2–0.7, n = 13). For comparison, the AP half width of excitatory neurons was on average 1.03±0.07 ms (range 0.4–1.6, n = 18). The firing patterns of 8 inhibitory neurons were tested in response to constant current pulses. Six neurons (presented in Fig. 2F–H, K) responded with high frequency (>200 Hz), non-adapting trains of action potentials and 2 (Fig. 2I, O) showed a “stuttering” response pattern with a lower maximal frequency (<124 Hz). As a group, inhibitory neurons had on average fewer primary dendrites (5±1.4, n = 8 vs. 6.7±2, P<0.027 Mann-Whitney U-Test) of slightly shorter length (114±39 µm, n = 8 vs. 147±24 µm, n = 20, P = 0.04 Mann-Whitney U-Test) than the excitatory neurons. In contrast, the axons of inhibitory neurons were denser and thus better preserved in the thin slice preparation than their excitatory counterparts, as is reflected by their larger total length (8619±4282 µm, n = 10 vs. 3872±1821 µm, n = 20, P<0.001 Mann-Whitney U-Test).

### Inhibitory Synaptic Connections

**Figure 3:**
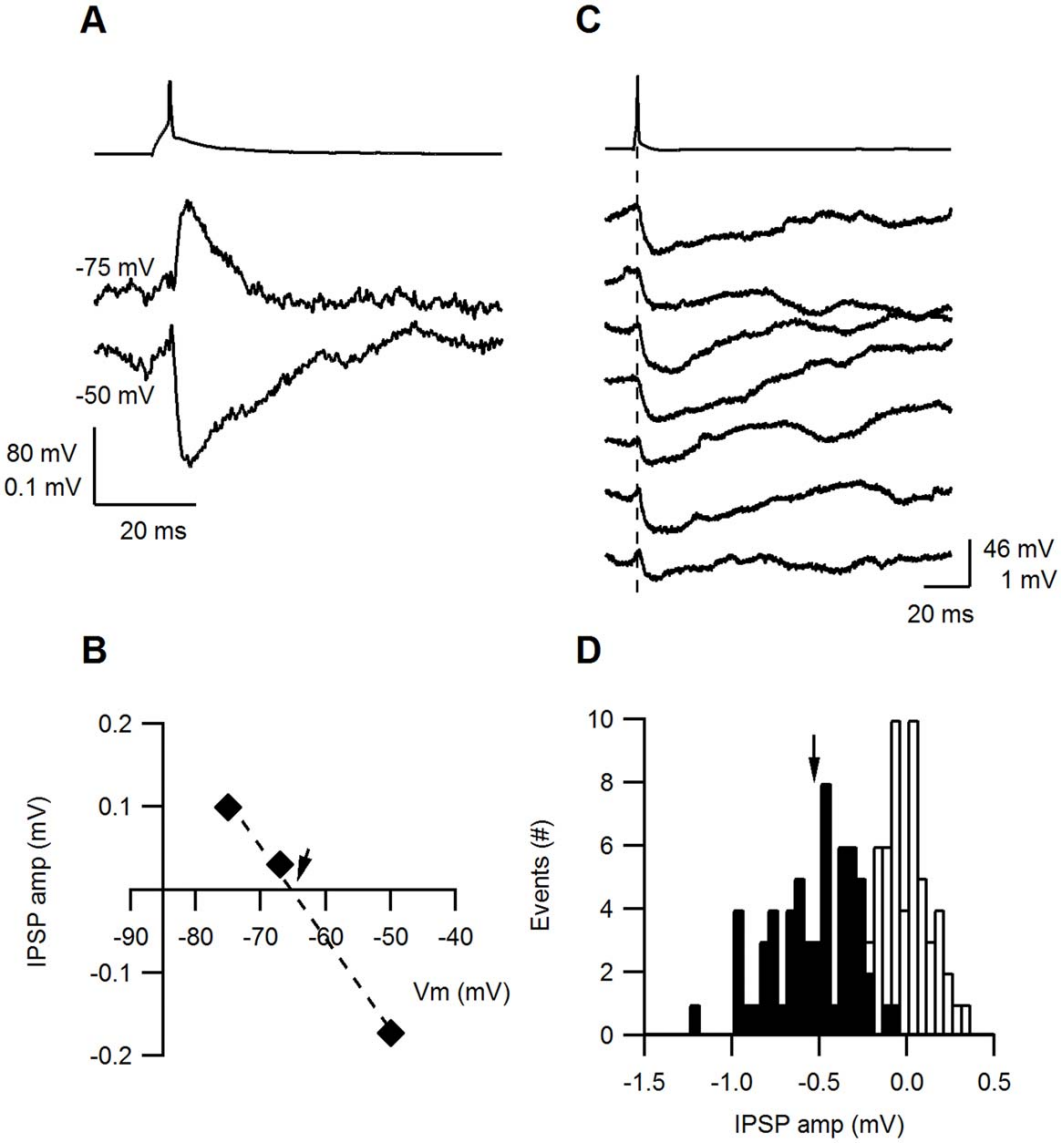
Inhibitory synapses. **A**. An AP evoked in the presynaptic inhibitory neuron and the simultaneously recorded IPSP (average of 15 traces, each) from the postsynaptic excitatory neuron at two membrane potentials. The IPSP was hyperpolarizing at a Vm of −50 mV but depolarizing at −75 mV. **B**. The IPSP amplitude plotted as function of the membrane potential (Vm). The data were fitted with a straight line (dashed) intercepting the x-axis (indicated by an arrow) at −65 mV. **C**. In 7 subsequent traces, IPSP amplitudes varied in response to single presynaptic APs, but no failures were observed. The postsynaptic Vm was held at −50 mV **D**. A histogram of the IPSP amplitudes (filled bars) was constructed from 60 traces and the background noise (empty bars). The IPSP histogram was fitted with a Gaussian distribution with a median at −0.53 mV (arrow) and a σ of 0.36 mV.

Fast IPSPs in the cortex are mainly mediated by GABA_A_ receptors forming Cl^−^ channels (reviewed in [29]. Due to a low intracellular versus high extracellular Cl^−^ concentrations ([Cl^−^]), the Cl^−^ reversal potential in most neurons is close to the resting Vm (Vrest), resulting in a small driving force for IPSPs at Vrest. To determine the IPSP reversal potential in our recording conditions, the postsynaptic Vm was held at values negative and positive to Vrest. Fig. 3A shows an example of the hyperpolarizing IPSP at Vm of –50 mV and the same IPSP producing a depolarizing potential from Vm of –75 mV. The IPSP reversal potential was calculated from the intercept of a linear fit of the IPSP amplitudes at 3 different Vm; in this experiment the reversal potential was –65 mV (Fig. 3B). The fast ionic diffusion between the large patch-pipette tip and the soma allows, in theory, the intracellular [Cl^−^] to be clamped to that of the pipette solution. Thus the IPSP reversal potential should match the Cl^−^ reversal potential calculated from the pipette and ASCF concentrations. In an attempt to lower the Cl^−^ reversal potential below the Vrest, we patched postsynaptic neurons with pipette solution containing either 4, 8 or 17 mM [Cl^−^]. The calculated Cl^−^ reversal potential of these solutions was −90 mV, −72 mV and –53 mV, respectively. Measured IPSP reversal potentials in different experiments ranged between –56 mV and –83 mV with an average at –69±9 mV (n = 8), but were not positively correlated with the theoretical Cl^−^ reversal potential (Correlations Coeff. = −0.37). These results indicate that either the [Cl^−^] at the dendritic synaptic locations is not well clamped by the pipette ([Cl^−^]), possibly due to dominant local Cl^−^ homeostasis at the dendrites, or, the depolarized IPSP reversal potential reflects a mixed flux of Cl^−^ and hydrocarbonate ([HCO_3_^−^]_i_) ions through the GABA_A_ receptor-linked channels [29]–[33], which are permeable to both anions. Close to the resting membrane potential, HCO_3_^−^ flows out of the cell and has thus a depolarizing effect, while Cl^−^ flows into the cell and hyperpolarizes the membrane potential.

### Size and Variability of IPSPs

In order to improve detection and measurements of IPSPs, the postsynaptic Vm was held at a depolarized value between –50 and –55 mV (on average −51±3 mV, n = 8). This was approximately 20 mV positive to the average IPSP reversal potential. Fig. 3C illustrates consecutive IPSPs from an exemplar I–E connection, which showed variable amplitudes between trials. The IPSP amplitude distribution was broad and skewed towards larger amplitudes (Fig. 3D, median = −0.53 mV and σ = 0.36 mV). The CV of the IPSP amplitudes in this experiment was relatively low (0.24) and no failures were observed (0/60 trials). The mean IPSP amplitude of all I–E connections was 0.598±0.4 mV (at –50 mV, n = 9) and the averaged CV was 0.295±0.12 (n = 9). In 4/9 experiments no IPSP failures were observed, and the averaged percentage failures over all experiments was 6±7.7% (n = 9). The single I–I connection we recorded from was similar in amplitude (0.78), CV (0.369) and F (5%) to the I–E connections.

### Size and Variability of EPSPs

**Figure 4:**
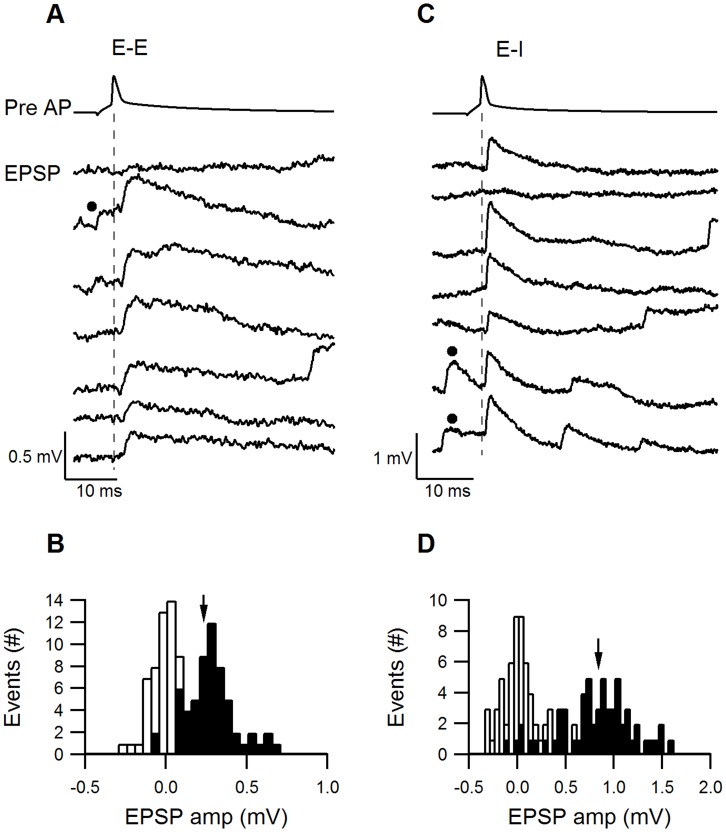
Reliability of E–E and E–I synapses. **A**. Fluctuations in the EPSP amplitudes at an E–E connection. The upper trace illustrates a presynaptic AP and the 7 subsequent traces show the response evoked in the excitatory postsynaptic cell. **B**. The EPSP amplitude histogram (filled bars) has a median at 0.24 mV and a σ of 0.152 mV. Black dots in A and B indicate spontaneously occurring EPSPs prior to stimulation. **C**. Fluctuations in the EPSP amplitudes at an example E–I connection. The upper trace illustrates a presynaptic AP and the 7 subsequent traces show the response evoked in the inhibitory postsynaptic cell. **D**. A broad distribution of the E–I EPSPs, with a median at 0.84 mV and a σ of 0.435 mV.

The size and variability of the EPSPs were determined from sets of consecutive trials evoked at slow stimulation frequencies (0.1–0.125 Hz). The postsynaptic Vm was −66±7 mV, n = 10 and −64±3 n = 8 for E–E and E–I synapses, respectively. An excitatory-to-excitatory connection (E–E) is illustrated in Fig. 4A. Unitary EPSP amplitudes were small and varied between trials and only occasionally was a failure observed (uppermost trial, failure percentage F = 10%). The EPSP amplitude histogram was fitted with a Gaussian distribution with a median of 0.23 mV and σ = 0.15 mV (Fig. 4B). In comparison, an excitatory-to-inhibitory connection (E–I) illustrated in Fig. 4C had larger EPSP amplitudes, but these were broadly distributed around the median of the Gaussian distribution (Fig. 4D, median 0.84 mV, σ = 0.43 mV) and exhibited occasional EPSP failures (second trial, F = 5%). The variability of synaptic responses calculated from their CV was similar for the E–E and E–I connections, as illustrated in Fig. 4A, C (CV = 0.34 and 0.4, respectively). On average, E–E and E–I EPSPs differed significantly in their mean amplitudes (0.38±0.35 mV, n = 11 and 1.54±1.55 mV, n = 8, respectively, P = 0.017 Mann-Whitney U-Test), but had similar CVs (0.424±0.155, n = 7 and 0.332±0.154, n = 7, respectively, P = 0.49 Mann-Whitney U-Test) and percentage failures (20±25%, range 5–74%, n = 7 and 17±28%, range 0–68%, n = 7, respectively, P = 0.21 Mann-Whitney U-Test).

**Figure 5:**
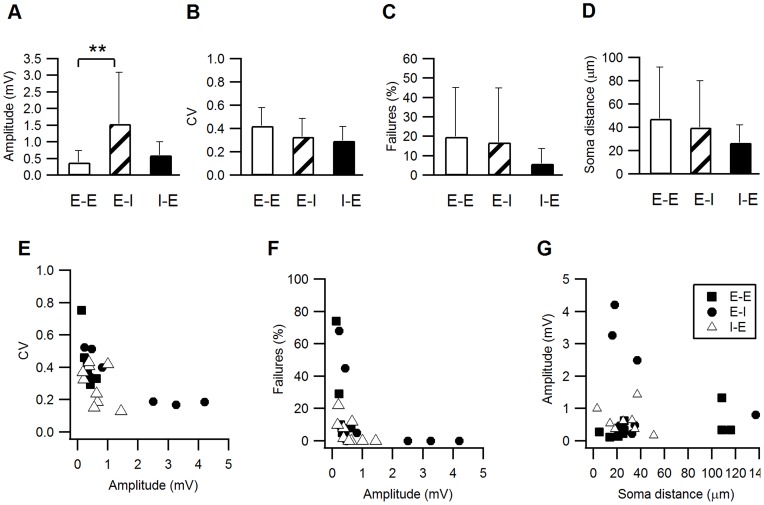
Variance analysis of E–E, E–I and I–E synapses. **A–C**. The averaged (±s.d.) PSP amplitude (**A**), CV (**B**) and percentage failures (**C**) were compared among the three connection types. The EPSP amplitudes of E–I connections were significantly larger than E–E EPSPs or I–E IPSPs (p<0.05). I–E connections had on average lower CVs and percentage failures, but these were not statistically significant. **D**. The average horizontal distances between the pre- and postsynaptic neurons were short for all connection types: E–E 47±14, n = 10; E–I 40±14, n = 8, I–E 27±5, n = 8. **E–F**. The CV and percentage failures of individual synapses were plotted against their average PSP amplitude. In, general, larger PSP amplitudes were correlated with lower CVs and percentage failures and smaller PSP amplitudes correlated with the higher CVs and percentage failure. Among medium-sized PSPs no obvious correlations with the CV or percentage failures were observed. **G**. PSP amplitudes were plotted against the horizontal distance between the pre- and postsynaptic neurons. No correlation could be observed between these parameters (cor. Coeff. = −0.08).

A comparison of the averaged size and variability of EPSPs and IPSPs is shown in Fig. 5 A–D and is summarized in Table 1. The most prominent difference between the synapses was that EPSP amplitudes of E–I synapses were significantly larger than E–E synapses (P = 0.017 Mann-Whitney U-Test). Although the CV and percentage failures of IPSPs (I–E) tended to be smaller than those of the EPSPs (E–E and E–I), these differences were not significant (P>0.1 Mann-Whitney U-Test).

We examined the relationship between the synaptic variability and amplitude of all individual synapses. The plot of CV against the PSP amplitude (Fig. 5E) shows that the highest CVs were measured in the smallest EPSPs and the lowest CVs were measured in the largest PSPs (E–I or I–E). However, most PSPs had amplitudes between 0.2–1 mV and within this range they were not correlated with the CV. The largest amplitude EPSPs and IPSPs had the lowest percentage failures (Fig. 5F) and commonly exhibited no failures at all, while the smallest PSPs exhibited the highest percentage failures. However, most of the PSPs between 0.2–1 mV (approximately 20 PSPs) had few failures (<10%) and did not obviously correlate with the PSP amplitude.

The PSP amplitude is a product of the number of release sites, the release probability (*p*) and the postsynaptic response, also termed the quantal size (*q*). The CV and failures plots suggest that very small PSPs might also have low release probabilities and fewer release sites, and conversely for the largest EPSPs. However, the fact that most of the PSPs at the mid-range amplitudes did not exhibit such a correlation suggests the involvement of postsynaptic factors such as the quantal size and electrotonic filtering. It is also possible that different determinants are involved at each connection type. To investigate this possibility further, we determined the number of contacts per connection based on the LM reconstruction of 15 reconstructed pairs of connected neurons (see last section). The number of contacts was small; on average 3, 2 and 5 contacts per E–E, E–I and I–E connection, respectively. Assuming these contacts to be actual release sites, and assuming identical *p* and *q* for each release site, we used the binomial model to calculate the release probability (see Methods), which was similar for all synapses (0.67±0.15, n = 7; 0.83±0.13, n = 7; 0.72±0.14, n = 9 for E–E, E–I and I–E, respectively). Thus, it seems likely that I–E connections tend to be more reliable because they are formed by a greater number of release sites. The quantal size (*q*) of the E–I synapses was much larger than that of the E–E or I–E synapses and was more strongly correlated with the E–I EPSP (0.97±0.79 mV, n = 7 Corr. = 0.99; 0.18±0.1, n = 7, Corr. = 0.44; 0.17±0.1 n = 9 Corr. = 0.73). These calculations suggest that the more dominant determinants of the PSP size appear to be the number of release sites and the quantal size. These calculations are, however, confounded by the inaccuracy of determining N from contacts identified in the light microscope and by the uncertainty in the adequacy of the quantal model for describing layer 4 synapses.

The PSP amplitudes were not correlated with the horizontal distance between the pre- and postsynaptic neuron somata (Fig. 5G, correlation coefficient −0.002), suggesting that the mean synaptic strength of a connection remains constant over the distances examined (<100 µm).

### Kinetics of Excitatory and Inhibitory Synapses

The kinetic properties of somatically measured PSPs are determined by the kinetics of the underlying synaptic conductance and by forward propagation of the PSP along the dendrites to the soma. Together, these determinants influence the total somatic charge contributed by the synapse, the time window in which it peaks, and hence strongly influence somatic summation and AP generation.

**Figure 6:**
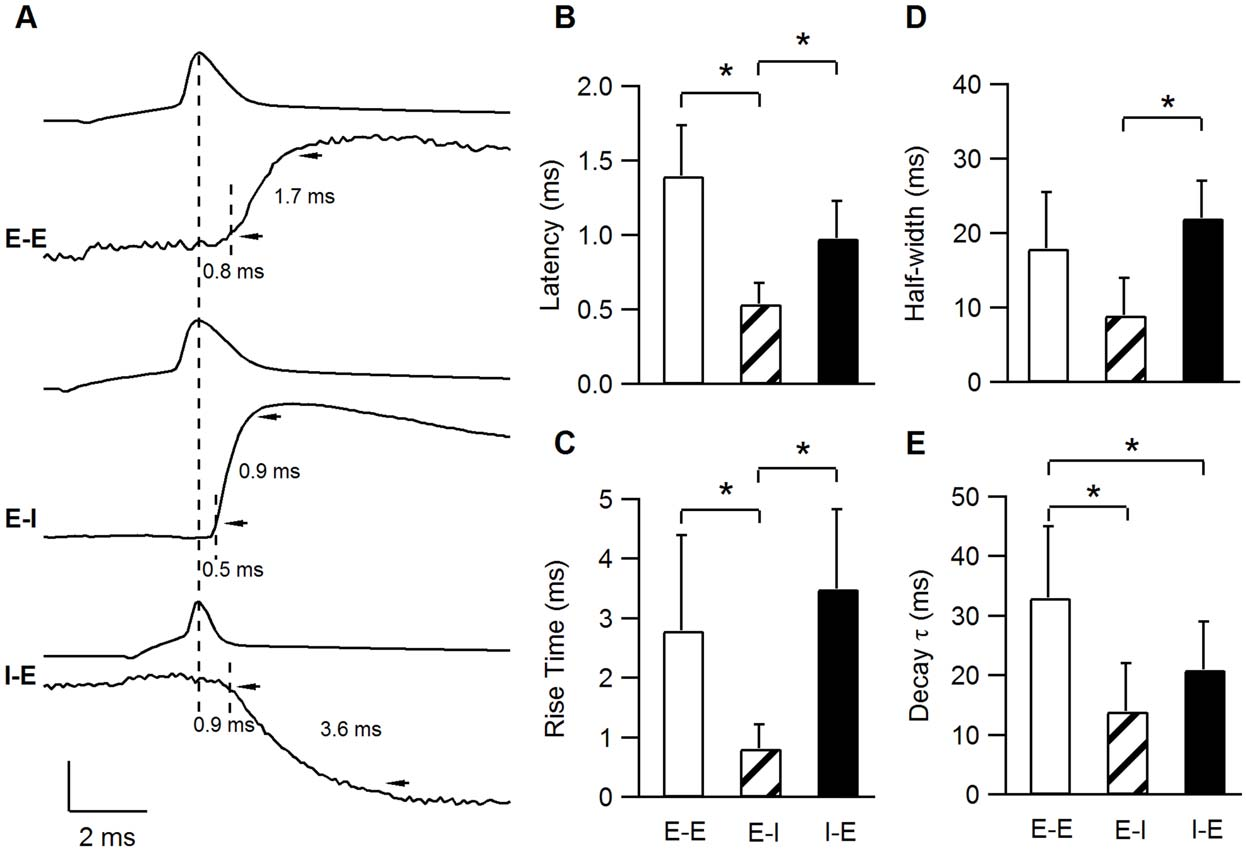
Synaptic Kinetics. **A**. Averaged PSPs from 3 individual connections illustrate the differences in synaptic kinetics of the various connection types. The presynaptic APs were aligned at their peak (depicted by the long dashed line). The short dashed lines on every PSP denote the time at which the PSP rose to 10%. The latency between the presynaptic AP peak and the 10% PSP was 0.8, 0.5 and 0.9 ms for the E–E, E–I and I–E synapses, respectively. The arrowheads point towards the 10% and 90% values of the PSP amplitude and the delay between them is defined as the rise time. Vertical scale bar represents potential and was 80 mV for all three APs and 0.13 mV, 0.85 mV and 0.2 mV in the E–E, E–I and I–E PSPs, respectively. **B–E**. E–I connections have significantly shorter latencies and faster rise times compared with E–E and I–E connections. All bar graphs represent the averaged values ±s.d.

Somatic PSP traces were aligned to the peak of the presynaptic AP and averaged PSP kinetic parameters, such as latency, rise time, decay time constant and half width were measured from the aligned-averaged PSPs. Aligned averaged PSPs from typical E–E, E–I and I–E synapses are plotted in Fig. 6 A. The time of the presynaptic APs peak (plotted above each PSP) and the time at which the EPSP reached 10% are depicted by dashed lines, arrowheads point to the 10% and 90% PSP amplitudes. Clearly, the E–I connection presented here had the shortest latency and fastest rise- and decay- kinetics (0.5, 0.9 and 16 ms, respectively). In comparison, the E–E EPSP and I–E IPSPs had longer latencies (0.8 and 0.9 ms, respectively) and slower rise times (1.7 and 3.6 ms, respectively).

The EPSP latencies (Fig. 6B) for the E–I connections were significantly shorter (0.54±0.14 ms, n = 7) than the E–E and the I–E connections (1.4±0.34 ms, n = 7 and 0.98±0.25 ms, n = 9, respectively, P<0.0001 Kruskal-Wallis U-Test). The mean rise time (Fig. 6C) of E–I synapses was the fastest at 0.82±0.4 ms (n = 7) compared with 2.8±1.6 ms (n = 7) for E–E EPSPs and 3.5±1.34 ms (n = 9) for I–E IPSPs (P = 0.001 Kruskal-Wallis U-Test). Although E–I EPSPs tended to have faster decay time constants and shorter half-width (Table 2, Fig.6 C, D), they were not significantly different from the E–E and I–E PSPs (P>0.1 Kruskal-Wallis U-Test. The single I–I IPSP recorded had slow onset and decay kinetics, similar to the I–E synapses. The postsynaptic membrane input-resistance (Rin), which also may influence the PSP rise- and decay-kinetics, was tested in a subset of the paired neurons and was similar for all connection types (Table 2, 73±38 MΩ n = 5, 90±20 MΩ n = 4 and 72±38 MΩ n = 3, for E–E, E–I, I–E respectively, P = 0.7 Kruskal-Wallis U-Test). Thus, the significantly faster onset kinetics of E–I synapses could not be accounted for by a lower somatic Rin value.

**Table 2:** EPSP/IPSP kinetics.

	Charge (mV*ms)	Latency (ms)	RT (ms)	Decay τ (ms)	HW (ms)	Rin post (MΩ)
**E–E**	12±17 (0.95–50)[n = 7]	1.4±0.34 (0.92–1.98) [n = 7]	2.8±1.6 (1.43–5.2)[n = 7]	33±12 (13–54) [n = 7]	20±7.5 (9–28) [n = 7]	73±38 (33–136) [n = 5]
**E–I**	20±22 (1–52)[n = 8]	0.54±0.14 (0.38–0.7) [n = 8]	0.82±0.4 (0.52–1.76) [n = 8]	14±8 (4–29) [n = 8]	9±5 (3–17) [n = 8]	90±20 (60–102) [n = 4]
**I–E**	16±15 (2–42)[n = 9]	0.98±0.25 (0.7–1.4) [n = 9]	3.5±1.34 (2–6.5)[n = 9]	21±8 (11–39) [n = 9]	22±5 (13–29) [n = 9]	72±38 (33–110) [n = 3]
**I–I**	35[n = 1]	1[n = 1]	3.44 [n = 1]	22 **[n = 1]	47 [n = 1]	21 [n = 1]

Latency, time from AP peak to 10% EPSP/IPSP; RT, rise-time 10–90%; Decay τ, single-exponential; HW, half-width; Rin, input resistance. Means are presented ± s.d,

** fitted with a α function with 2exponents. Ranges from min to max values are written in parentheses and number of observations in square brackets.

Most surprising was the difference in kinetics between the E–E and E–I synapses, which are formed by the same presynaptic neurons. This raises the possibility that postsynaptic parameters are critical in creating this difference. The two parameters likeliest to affect the EPSP kinetics are the synaptic conductance and the synaptic location. A faster synaptic conductance could mediate the E–I EPSPs, as previously demonstrated in other E–I synapses [34], [35] and thereby reduce the E–I EPSP rise and decay times. Shorter electrotonic distances of the E–I synapses on the postsynaptic dendritic tree would reduce the transmission time of the EPSP to the soma and also sharpen the EPSP measured at the soma [36]. The overall dendritic branching pattern and the local dendritic morphology at the synaptic location would also shape the local dendritic EPSP and therefore its waveform in the soma [37], [38]. To test this possibility we simulated EPSP propagation in realistic dendritic trees of the postsynaptic excitatory and inhibitory neurons in E–E and E–I synapses, based on our detailed 3D reconstructions. We asked whether a different morphoelectrotonic distance of these synapses can explain their kinetics and whether the underlying synaptic conductance also needs to be different.

### Compartmental Simulations of EPSPs onto Smooth and Spiny Stellates

We used simulations to explore whether dendritic attenuation and synaptic location might account for the fast recruitment and kinetics of the E–I synapses. The morphologies and passive properties of six excitatory (5 spiny stellate cells and a star pyramid) and four inhibitory (basket) neurons were used as templates for these simulations. Our general approach was to try to model the experimental data as closely as possible by using only those neurons for which we had the full data set of morphological and passive biophysical properties. We attached a single excitatory synapse successively to each dendritic compartment and measured the simulated EPSP evoked at the soma.

**Figure 7:**
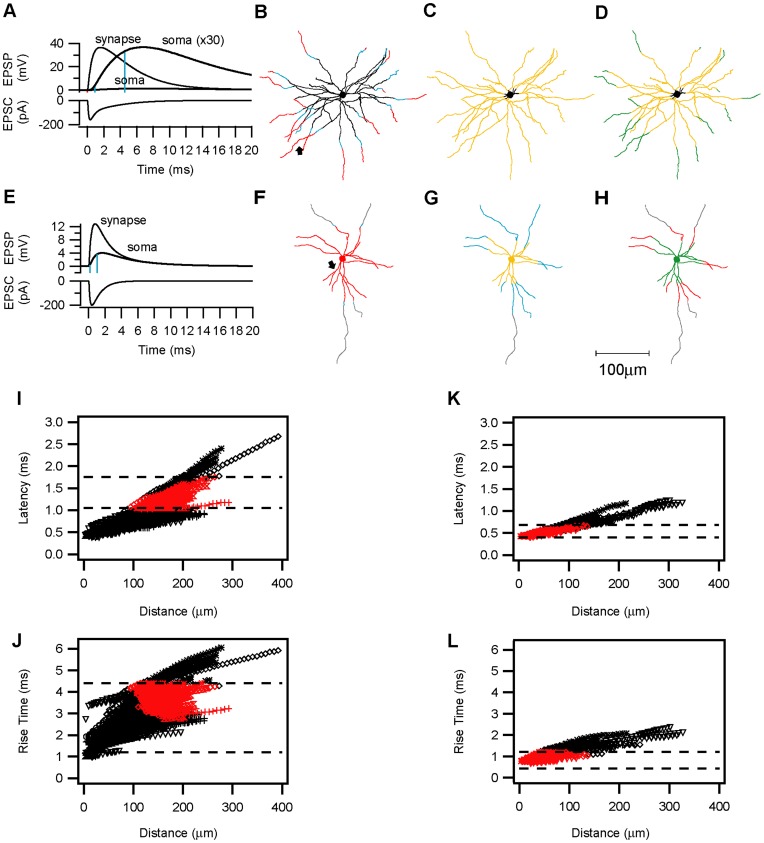
Compartmental model simulations of synaptic kinetics. Electrotonic attenuation of the synaptic current along the dendrites en route to the soma, results in smaller and slower somatic EPSPs. **A, E**. A single synaptic contact with a conductance of 3.8 nS was simulated on (**A**) a spiny stellate or (**E**) a basket cell dendrite at the location where a putative contact was identified (arrows in **B** and **F**, respectively). The decay time constant (τ2) of the conductance in **E** was faster than in **A** (1 ms vs. 2.3 ms). The simulated EPSCs and the resulting EPSPs at the synapse are drawn in a thin line. The same EPSPs measured in the soma are drawn with a thick line. In (**A**) the somatic potential trace was multiplied (×30) to allow direct comparison of the kinetics. The 10% and 90% EPSP amplitudes are marked by the blue lines. Note that the more distal contact simulated in **A** is more strongly delayed, slowed and attenuated than the proximal contact simulated in **E**. **B–H**. A single synaptic activation was simulated successively in all compartments of the spiny stellate (**B–E**) and smooth basket (**F–H**) neurons. In each compartment, the latency of the EPSP arrival at the soma and the somatic 10%–90% EPSP rise times were analyzed and compared with the experimentally observed values. The somatic and dendritic compartments were then colored according to the following categories: Black: simulated values lower than the smallest experimentally observed latency or rise time. Grey: simulated values larger than the largest experimentally observed latency or rise time. Blue: simulated values within the range between the smallest and the largest observed values. Red: Mean EPSP latency (average±s.d.). Yellow: Mean EPSP rise time (average±s.d.). Green: Overlapping compartments in which both rise-times and latencies were within the average range. **B, F**. Latency plots. **C, G**, Rise time plots. **D, H**, overlap plots of latencies and rise times. The scale bar is 100 µm. **I–L**. Simulations were repeated in 5 spiny stellate cells and 1 star pyramid (**I, J**) and 4 basket cells(**K, L**) neurons, each presented by a different symbol. **I, K**, Somatic EPSP latencies plotted against the dendritic distance from the soma for all spiny neurons (**I**) and basket cell (**K**) dendrites. **J, L**, Plots of the rise times at different dendritic distances from the soma. The dashed lines represent the experimental values of the corresponding average+s.d. Red symbols represent the overlap compartments for the latency and rise times in each neuron. Spines in **A–D, I–J**, were incorporated by scaling Cm and Rm in the dendrites. A faster conductance (as in E) was simulated in **F–H, K–L**.

Examples of the simulated EPSC and EPSPs evoked at the synapse (indicated by an arrowhead in the lower cell plot) and at the somata of the spiny stellate cell (7A) and the smooth basket cell (7E), respectively. Although in both cells the amplitudes of the somatic EPSPs were attenuated and their waveform slowed, this effect was far more pronounced for the distal synapse on the spiny stellate neuron. The somatic EPSP latency and rise time were analyzed and plotted against the dendritic location (distance from soma). Those dendritic compartments in which the experimentally measured average EPSP latencies and rise times were reproduced, were colored red and yellow, respectively. Compartments where both parameters were simultaneously satisfied were colored green. Compartments in which the latencies or rise times were within the experimentally observed range (min-max) were colored blue, black and grey represent values either below (black) or above (grey) the measured ranges. In the example spiny stellate neuron plotted in Fig. 7B–D only distal compartments yielded somatic EPSPs with experimentally-observed latencies (Fig. 7B). However, rise times within the experimental range could be evoked from the entire dendritic tree, with the exception of the most proximal dendritic branches and the soma (Fig. 7C). The green overlap regions (Fig. 7D) were thus distal and determined by the latencies. In contrast, latencies within the experimentally observed range for E–I synapses were evoked from the soma and the proximal (100 µm) dendrites in the smooth basket cell plotted in Fig. 7F. EPSP rise times within the experimentally observed range could only be evoked when a faster synaptic conductance was simulated. Even with a faster synaptic conductance, only very proximal dendritic compartments yielded sufficiently fast somatic-EPSPs (Fig. 7G). The overlap region for this inhibitory neuron is thus constrained by the rise times to the proximal compartments (Fig. 7H).

**Figure 8:**
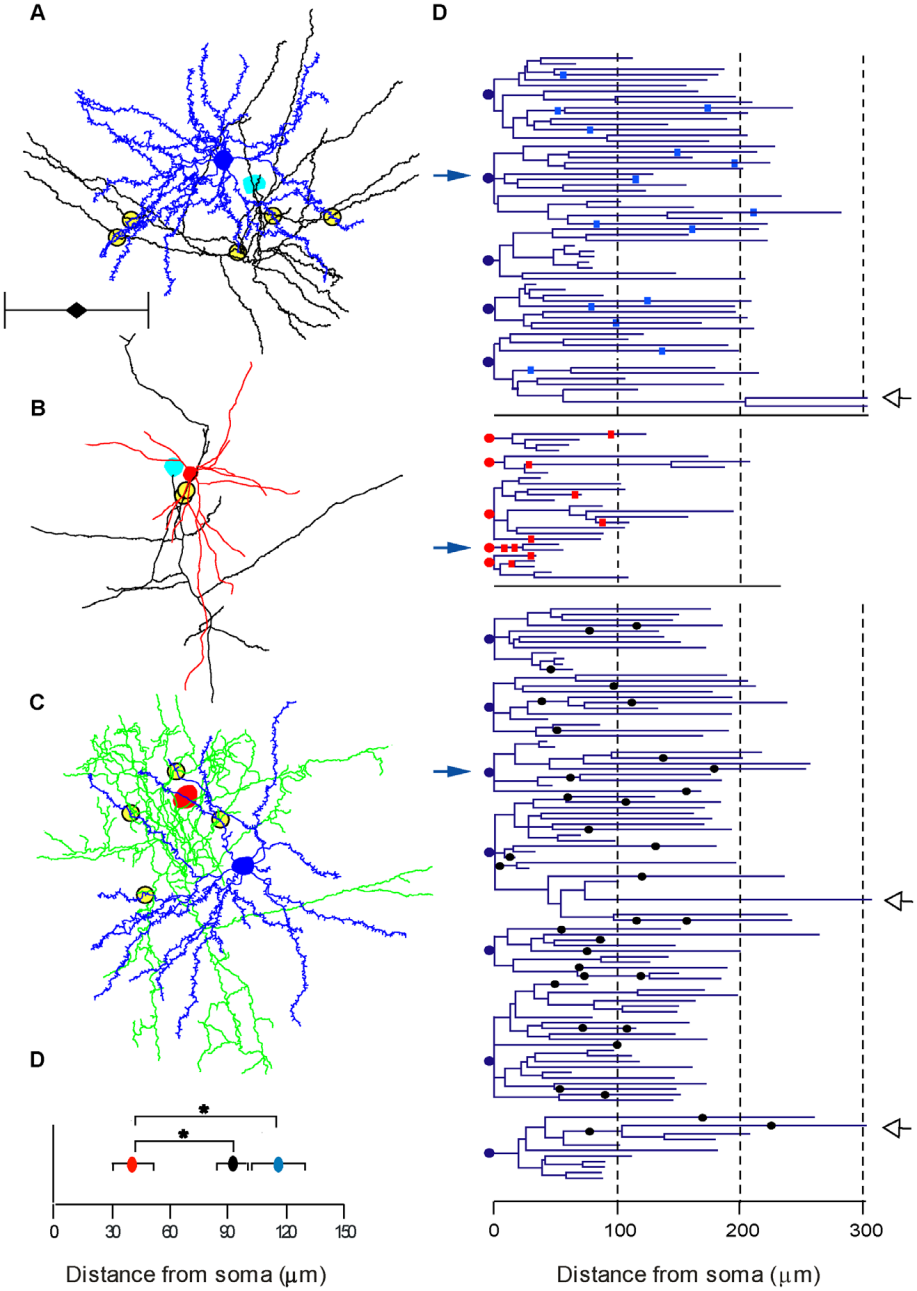
Location of LM contacts. Contact sites seen at LM between the presynaptic axon and the postsynaptic dendrites were determined for all reconstructed pairs. **A–C** illustrates examples of an E–E, E–I and an I–E pair. The presynaptic soma of the spiny stellate cell is colored cyan and the axon is black. The basket cell’s soma and dendrites are colored red and the axon green. Postsynaptic spiny stellate cell is colored blue. For clarity, axons were trimmed to a sphere of about 400 µm diameter around the soma, but complete dendritic trees are presented. Putative contacts are marked by yellow-filled circles. Scale bar is 200 µm. **D**. Dendrograms of excitatory cells (somata indicated by blue circle) and inhibitory cells (somata indicated by red circle). Position of contacts on the dendrites are indicated by cyan (E–E) and red (E–I) rectangles. I–E contacts are indicated by black circles. Open arrowheads indicate three apical dendrites clipped at 300 µm **E**. The averaged distance of E–I contacts was significantly lower than E–E and I–E (P<0.05).

These simulations were repeated in additional neurons, showing that latencies in the basket cells (Fig. 7K) (average: 0.647±0.19 ms, range 0.425–1.25 ms, n = 638 compartments) were slightly shorter than in the spiny neurons (Fig. 7I) (average: 0.875±0.343 ms, range 0.4–2.675 ms, n = 2733 compartments, compartments of a single apical dendrite >400 µm were omitted for presentation purpose). The EPSP rise times in the basket neurons (Fig. 7L) were faster than in the spiny neurons (Fig. 7J), mainly due to the faster kinetics of the underlying simulated synaptic conductance (See Methods for details). When the same “slow” synaptic conductance was simulated in the spiny and smooth neurons, the same range of rise times was observed in both cell types. In the spiny neurons (Fig. 7I, J), compartments satisfying both the latency and rise time requirement of the E–E connections (“overlap” compartments) were located more distally (mean 163±33 µm, range 94–291 µm, n = 458) than the overlap compartments of the smooth basket neurons (Fig. 7K, L), which reproduce the E–I kinetics (mean 49±26 µm, range 3.5–136 µm, n = 312, P<0.0001, T-test). This difference between the smooth and spiny neurons did not depend on the existence of spines in the latter, since simulations performed without scaling for spines, yielded nearly identical latencies and rise times in the spiny neurons. These simulations suggested that a proximal versus distal location of the E–I and E–E synapses, respectively, is most likely to explain the differences in their kinetics, rather than the different morphology or local passive membrane properties of spiny and smooth basket cells.

**Figure 9:**
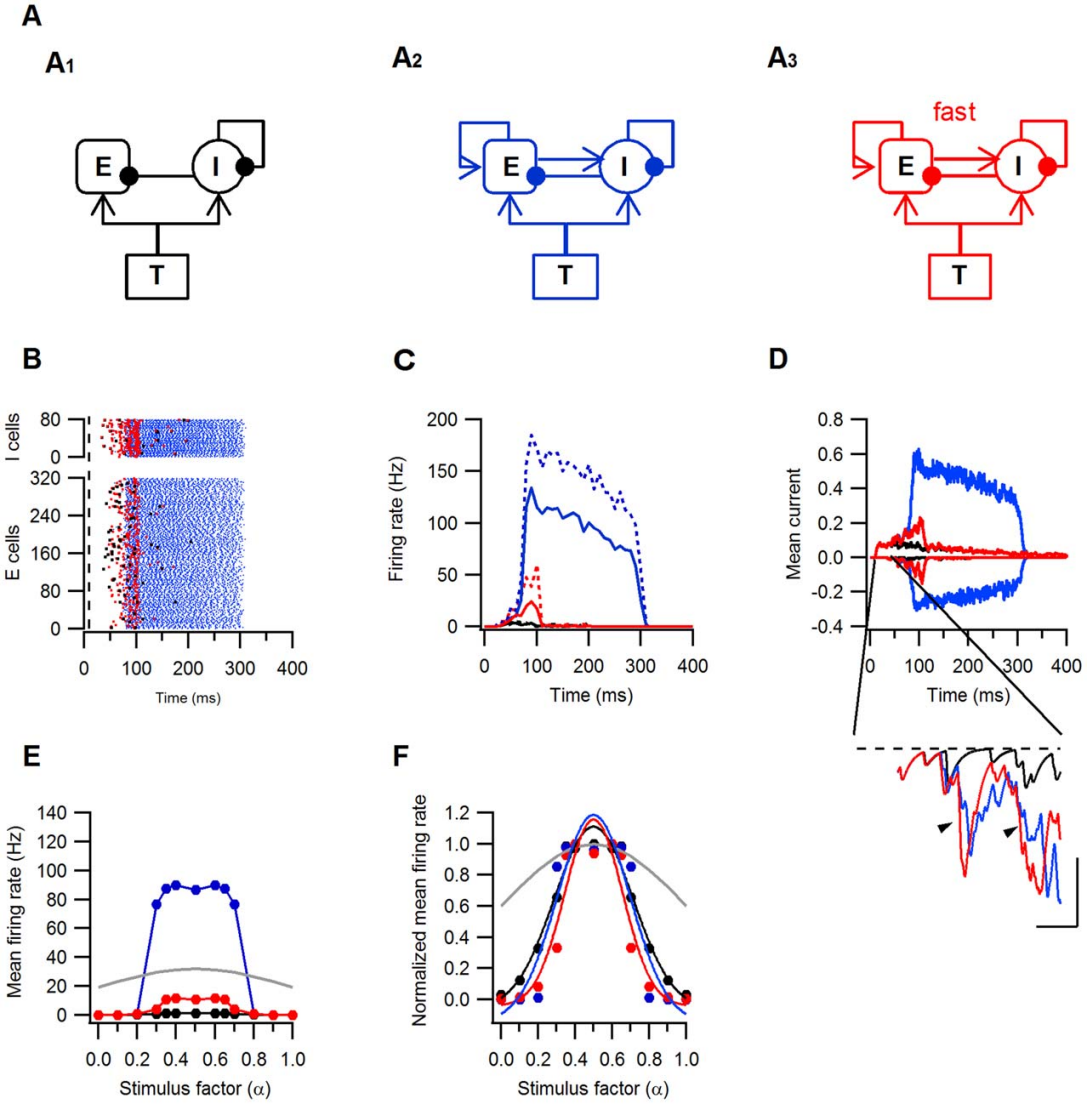
Effects of fast E–I recruitment on the activity and tuning curves of a model recurrent L4 network. Three models were used to simulate activity in the recurrent network. All models included excitatory (E) and inhibitory (I) L4 neurons and feedforward thalamic (T) input synapses and were identical in all but the recurrent excitatory synapses. Excitatory and inhibitory synapses were drawn with arrows and filled circles, respectively. **A1**. The feedforward model in which E–E and E–I synapses were silenced. **A2**. The second model incorporating recurrent E–E and E–I synapses with identical kinetics and latencies. **A3**. The third model is structurally identical to A2 but E–I synapses have faster kinetics and shorter latencies. Models 1, 2 and 3 and all the results are colored black, blue and red, respectively. **A–D**, Responses of all three models to the same feedforward synaptic input (Fin = 29.5 Hz). **B**. Raster plots of the excitatory (E) and inhibitory (I) spike times, dashed line marks stimulus start. **C**. Peri-stimulus histogram of the firing rates of the excitatory (solid lines) and inhibitory (dashed lines) neurons binned at 10 ms intervals. **D**. Mean excitatory (positive to 0) and inhibitory (negative to 0) currents to the excitatory L4 neurons. Current is given in arbitrary units. The inset shows the mean inhibitory current between t = 40 and 80 ms from simulation start. The arrowheads point towards the earlier onset and faster rise of the current in model 3 compared to model 2. Scale bars are 10 ms and 0.03 I. **E–F**. Tuning curves of the excitatory neurons. **E**. Mean firing rates as a function of the stimulus parameter α, note the many-folds amplification of the responses in models 2 (blue) and 3 (red) in respect to model 1(black). **F**. The mean firing rates normalized to the maximal response as a function of α. The lines represent the best Gaussian fits to the normalized responses. The grey line is the normalized input frequency. Note the narrowing of the tuning curve in model 3.

### Putative Synaptic Location

The compartmental simulations supported the hypothesis that the reason for the fast recruitment of E–I synapses was their proximity to the soma. To assess this possibility we reconstructed 15 pairs of neurons making an E–E, E–I or I–E connection (5 of each type) and identified in the light microscope the sites of contacts between the presynaptic axons and the postsynaptic dendrites, examples of which are shown in Fig. 8A–C. We identified a total of 15 E–E, 9 E–I and 36 I–E contacts (Fig. 8D–F), whereby the relatively small number of E–I contacts reflects the incomplete morphological recovery of inhibitory dendrites. On average E–E contacts were located on higher order dendrites compared with E–I and I–E contacts (4^th^, 2^nd^ and 3^rd^, respectively, range for all was 1^st^ –5^th^ order). The I–E contacts made by basket cells were in all but one case found on basal and apical dendrites, but not on the soma of the target cell. The E–I contacts were located at an average distance of 41±33 µm from the soma (n = 9, Fig. 8G). In contrast, E–E and I–E contacts were located significantly (p = 0.002, Kruskal-Wallis U-Test) more distally at average distances of 116±54, n = 15 and 92±47 µm n = 36, respectively (Fig. 8G).

We measured the axonal distance from the presynaptic cell soma to the contact on the postsynaptic dendrite and found that the axonal distance of the presynaptic boutons were not different between the EE and EI synapses (196±80 µm n = 13 and 237±147 µm n = 9, respectively, P = 0.7 Mann-Whitney U-Test).

Given the dendritic locations of the various connections we could now estimate the synaptic conductances underlying each PSP, assuming there was a synapse at the site of contact (See Methods for details). The synaptic conductance was on average: 0.57 nS, 0.34 nS and 2.06 nS per contact of the E–E, E–I and I–E connections. Comparison of the synaptic conductance values to the measured somatic PSP amplitudes emphasizes the impact of the synaptic location on the somatic voltage and hence on the neuronal output. On the other hand, local excitatory/inhibitory interactions at the dendrites would be sensitive to the relative size and timing of the conductances which implies that under some conditions the I–E connections could exert strong shunting inhibition at the dendrites despite a seemingly small postsynaptic hyperpolarization at the soma [39].

### A Recurrent Network Model with Fast E–I Synapses

Can the 2–3 ms-faster recruitment of E–I synapses bear significant implications for the neural activity in the highly recurrent network of visual cortex L4?

To address this question, we generated a model recurrent network composed of excitatory and inhibitory LIF neurons driven by thalamic input (Fig. 9A). We compared three versions of this model: First, a feedforward model in which E–E and E–I synapses were silenced but T-E, T-I, I–E and I–I were activated (Fig. 9A1). A second model, in which recurrent excitatory synapses were activated and excitatory recurrent synapses (E–E, E–I) had the same rise and decay kinetics and the same activation latencies (Fig. 9A2). The third model, was similar to the second but E–I synapses were modified to have faster kinetics and shorter latencies, reflecting our experimental findings (Fig. 9A3). Raster plots of neurons spike times (Fig. 9B) and the corresponding mean firing rates (Fig. 9C) during the simulation, show that activation of equal recurrent excitatory synapses (model 2, blue lines and symbols) strongly increased the number and rates of spikes generated in response to the feedforward input. In the second model (red lines and symbols), recurrent excitation also resulted in amplification of the feedforward input. However, this amplification was weaker and its duration shorter than in the third model due to the earlier onset and peak of the inhibitory current in the third model. (Fig. 9C, inset). Thus the simulations show that fast recruitment of E–I synapses provides a powerful mechanism for entrainment of activity in a recurrent network while allowing for amplification of the feed forward input.

As neurons in the visual cortex are tuned to various stimulus parameters, such as orientation, we next examined the neurons tuning function in each of the network models. The frequency of the feedforward input to both excitatory and inhibitory neurons followed a Gaussian-like tuning function (refer to Methods for calculations). Recurrent excitatory connections (models 2 and 3) amplified the output firing rates over most of the tuning function (Fig. 9E). The tuning bandwidth however, was narrowed only in model 3 (Fig. 9F). Thus fast E–I synapses, enabled amplification and sharpening of the tuning response curve simultaneously.

Theoretical and experimental work suggested that intracortical inhibition can control the activity in the excitatory recurrent network and modify its input-output function [14], [40]–[45]. The simple models which we used demonstrate that fast recruitment of E–I synapses can be an effective way of achieving this balance and enhancing visual responses while restoring or even enhancing the tuning of the feedforward input.

## Discussion

### Network Connectivity

Excitatory neurons in this study were classified as star pyramidal or spiny stellate cells according to their dendritic trees, while inhibitory neurons (all of which had fast action potentials), were classified mainly by their axonal morphology as basket cells. Interestingly, synaptic pairs involving slow or regular firing inhibitory neurons (of bipolar morphology) were not found, in contrast to their apparent abundance in layer 4 of the rat [6], [46], [47]. This may reflect the scarcity of such inhibitory neurons in cat layer 4. Most of the basket cells we recorded closely resemble a specific subgroup of small basket cells of layer 4 termed “clutch cells” [48], which have axons that form a dense local axonal cluster.

Previous studies of layer 4 in cat visual cortex [6], [7], [9]–[13], [49] and in rodent [18], [46], [47], [50]–[53] revealed a strong recurrent connectivity within the excitatory population and between excitatory and inhibitory neurons. The general view emerging is that in the cat most of these synapses produce moderate-to-large and reliable PSPs, and that excitatory connections onto fast spiking inhibitory neurons are largest in amplitude [6], [12], [46]. Inhibitory synapses tend to have the lowest variability, probably due to the multiple contacts that comprise each connection [10], [11]. Our electrophysiological data are in good agreement with these reports and expends on them by revealing novel properties of the synaptic kinetics and timing. Our morphological data and simulations extend previous reports to show that the number of synapses per connection and their dendritic location contribute strongly to the differences in synaptic strength among the various connection types in layer 4.

### Synaptic Properties

#### Synaptic strength and variability

The E–E connections we recorded with the patch clamp technique have lower amplitudes and higher CVs compared with the same type of connections previously studied in thick slices (400–500 µm) with sharp pipettes [7], [9], [13]. While the dendritic trees of spiny stellate and pyramidal neurons are largely conserved, in the thinner slices we used (300 µm) the axons are more severely truncated. It is therefore likely that the number of contacts per connection in the E–E pairs is smaller in our study. Consistent with this interpretation is our observation that the amplitudes and CVs of E–I and I–E connections recorded were comparable with those previously reported in thick slices [12], suggesting that the closest range connections (E–I and I–E) are relatively better preserved in the thin slices we used. The release probability was high, in agreement with previous studies [7], [9], [13].

#### Synaptic Kinetics

Patch clamp recordings allowed us to perform detailed and precise measurements of the PSP kinetics. The results show that E–I synapses have the fastest rise- and decay kinetics and shortest latencies. The PSP rise and decay times are determined by the synaptic conductances, their electrotonic distance and by the membrane time constant (τm). Given that τm of all cell types was similar, it is unlikely to be the reason for the faster kinetics of E–I synapses. While the electrotonic location of the synapses can strongly affect their kinetics, our simulations indicated that only a fast conductance could mimic the kinetics of the experimentally measured E–I EPSPs, even at the closest distances from the soma. Previous studies in rat neocortex had shown that EPSPs onto fast spiking inhibitory neurons are mediated by AMPA receptors containing the GluR1 and GluR4 subunits, which have particularly fast kinetics and are calcium-permeable [34], [54], [55]. Immunostaining of glutamate receptor (GluR) subunits in the cat visual cortex showed a prevalence of GluR1 subunits in inhibitory neurons of layers 2–5 [56], suggesting that they could mediate the fast EPSPs we observed in the inhibitory neurons. In contrast, the slower kinetics of the E–E synapses could reflect a higher content of slow-gating NMDAR-channels [51], [57] and the existence of GluR2/3 subunits of the AMPA receptors [56]. In addition, the distal location of these synapses significantly contributes to extending the time course of the EPSP, as is expected from theoretical work [36], [58], [59] and is shown by our own simulations.

Inhibitory neurons in cortex are GABAergic and their postsynaptic targets express mainly GABA_A_ receptors [60] forming fast-gating channels [29], [30], [61]. GABA_A_ IPSCs rise within <1 ms and decay within less than 10 ms [62]. The I–E synapses we recorded are most likely to be of the GABA_A_ type, based on their neural morphology, Cl^−^ sensitivity and kinetics. Thus the synaptic conductance underlying E–I synapses might be faster than those mediating E–E and I–E synapses, which have similarly slow kinetics. The difference in PSP latencies, however, could not be accounted for by the various conductances, but were likely determined by the dendritic location of the synapses. Presynaptic parameters such as axonal conduction time and the synaptic delay to transmitter release could, in theory, contribute to the PSP kinetics and latencies (reviewed in [63]. However, axonal conduction time is unlikely to underlie the differences between the E–E and E–I connections, since these are located at similar distances along the presynaptic excitatory axons. Synaptic delays at high release-probability synapses are fast (0.1–0.3 ms) [55], [64]–[66] and thus cannot account for the 1 ms latency differences between the E–I and the E–E synapses.

Our simulations, buttressed by the morphological analysis of putative synaptic contacts, suggest that excitatory synapses are located on distal portions of other excitatory neurons, but on more proximal dendrites of inhibitory cells. Exactly this distribution was previously shown in our anatomical studies of spiny stellate cells [67] and small basket cells [68] in layer 4 of cat visual cortex. Inhibitory neurons in layer 4 of cat [48] and monkey [69] visual cortices, specifically target dendritic shafts and spines of excitatory neurons. Consistent with these studies, our morphological analysis showed that the axons of the basket cells contacted the distal dendrites of neighboring spiny cells. Functionally, it implies that inhibitory synapses on spiny neurons in layer 4 locally oppose excitatory synapses rather than exert, primarily, a peri-somatic inhibition of the summed dendritic excitation. Dendritic propagation of IPSCs mediated by the fast GABA_A_ conductance prolongs their duration at the soma and hence converts them into slow hyperpolarizing currents. This property is considered important for explaining direction selectivity in the visual cortex, which was so far assigned to the slow GABA_B_ conductance [70]. However, experimental evidence shows that pharmacological blockade of GABA_A_ but not GABA_B,_ reduces or abolishes direction selectivity [71]–[73]. Hence, the existence of distal dendritic inhibition with its slow somatic time course might help resolve this discrepancy.

### A Sensitive Dynamic Control of Recurrent Excitation

A number of theoretical studies predict that in order to prevent run-away recurrent excitation, the total inhibitory conductance has to be several fold larger than the excitatory conductance [14], [70], [74], [75]. Although single inhibitory synapses may indeed have a larger conductance than excitatory ones, there are fourfold more excitatory synapses. This places constraints on the ability of inhibition to control excitation in strongly recurrent networks. Our simulations show how the fast recruitment of E–I synapses eases these constraints and allows even smaller inhibitory conductances to control excitation. Numerous studies have demonstrated that local inhibition profoundly changes the amplitude of responses to visual stimuli in the cortex [41]–[45], [71]–[73]. Some of these studies have also shown that local inhibition can modify the response curves of cortical neurons to visual stimuli. Broadening of orientation tuning curves and reduction of direction selectivity has been reported [42], [44] after block of inhibitory synapses. Our network simulations show that inhibition can exert both of these actions: response gain control and sharpening of response tuning curves, if it is rapidly recruited by the recurrent cortical network.

Our data suggest that spatial and temporal properties of excitatory and inhibitory synapses in layer 4 of the visual cortex may be precisely tuned to provide an efficient modulation of the recurrent network. However, the possible roles *in vivo* of the precise localization and timing of these synapses, their involvement in the construction of receptive field specificity, including basic features like orientation and direction selectivity and contrast invariance, remain to be examined in networks models based on realistic biophysical measurements.

## Supporting Information

Appendix S1 **Differential equations for the Leaky Integrate & Fire neurons of the network models.** Excitatory and inhibitory neurons were modeled as current-based leaky integrate-and-fire (LIF) point neurons receiving three types of inputs: thalamic (T–E or T–I), excitatory (E–E, or E–I) and inhibitory (I–E or I–I). Each synapse is described by a double-exponential α function with distinct kinetic parameters. The Appendix describes the differential equations of the synaptic currents.
(DOCX)
