# Supplementary material for: Fast Recruitment of Recurrent Inhibition in the Cat Visual Cortex
Source: PLoS One. 2012 Jul 25;7(7):e40601. doi: 10.1371/journal.pone.0040601 (PMC3405110; doi:10.1371/journal.pone.0040601)
Supplement: Appendix S1 — Differential equations for the Leaky Integrate & Fire neurons of the network models. Excitatory and inhibitory neurons were modeled as current-based leaky integrate-and-fire (LIF) point neurons receiving three types of inputs: thalamic (T–E or T–I), excitatory (E–E, or E–I) and inhibitory (I–E or I–I). Each synapse is described by a double-exponential α function with distinct kinetic parameters. The Appendix describes the differential equations of the synaptic currents. (DOCX) [file pone.0040601.s001.docx]

# Appendix S1

**Appendix for network simulations**

Excitatory and inhibitory neurons were modeled as current-based leaky integrate-and-fire (LIF) point neurons (modified from NEURON’s IntFire4 mod file [21–23]. This model uses an event-based integration scheme in which the subthreshold membrane potential of each neuron is analytically calculated and its firing times are estimated by Newton iteration. Synaptic events (positive and negative) are modeled as double-exponential input currents each adding a weight µe or ωi to the total excitatory or inhibitory current variables, respectively. The total currents are normalized and act directly on the membrane potential. Excitatory LIF neurons received 3 types of synaptic inputs: thalamic (T-E), excitatory (E-E) and inhibitory (I-E). Inhibitory LIF neurons also received 3 types of synapses: thalamic (T-I), excitatory (E-I) and inhibitory (I-I). Each of the incoming inputs was added to its own total current.

The membrane potential is calculated between 0-1, where 0 is equivalent to the resting membrane potential and 1 to the AP threshold. When m reaches a value of 1 the neurons ‘fires’ and the membrane potential is reset to 0.

The membrane potential (m) is described by the following differential equation:

1.1

$$\frac{dm}{dt}=-\frac{1}{\tau_{m}}m+\sum_{T} B_{ta}T2\sum_{e} B_{E}E2+\sum_{i} B_{I}I2$$

Where τ_m_ is the membrane time constants and $B_{ta}$, $B_{E}$ and $B_{I}$ are the normalization factors for the thalamic (T2) excitatory (E2) and inhibitory ($I2$) currents, respectively.

In every iteration of the model, the currents and the membrane potential are calculated as follows:

1.2

$${T1}^{'}=-\frac{T1}{\tau_{T1}}$$

1.3

$${T2}^{'}=-\frac{T2}{\tau_{T2}}+A_{ta}T1$$

1.4

$${E1}^{'}=-\frac{E1}{\tau_{E1}}$$

1.5

$${E2}^{'}=-\frac{E2}{\tau_{E2}}+A_{e}E1$$

1.6

$${I1}^{'}=-\frac{I1}{\tau_{I1}}$$

1.7

$${I2}^{'}=-\frac{I2}{\tau_{I2}}+A_{I}I1$$

1.8

$$m^{'}=-\frac{m}{\tau_{m}}+B_{ta}T2+B_{E}E2+B_{I}I2$$

Equations 1.2, 1.4 and 1.6 describe the closed states of the input currents while equations 1.3, 1.5 and 1.7 describe their open states. The variables τ_T1_ τ_E1_ and τ_I1_ describe the rise time constants of the thalamic, excitatory and inhibitory synapses, respectively. The decay time constant are described by τ_T2_ τ_E2_ and τ_I2_, respectively. The variables A_ta_, B_ta_, A_e_, B_E_, A_i_, and B_I_ are normalization factors of the respective synaptic inputs. They are chosen such that an isolated excitatory synaptic input of a weight ωe=1 produces a maximal current E of 1 and a maximal m of 1. An isolated inhibitory input of a weight ωi =-1 produces a maximal current I of -1 and a maximal m of -1. We calculated the following factors for a τ_m_ =20 ms; the thalamic synapse: A_ta_=5.615, B_ta_=0.44616, for the E-E and the “slow” E-I synapses A_e_=5.615, B_E_=0.44616, for the fast E-I synapse A_e_=6.744, B_E_=0.75598, for the I-E synapse A_i_=5.3813, B_I_=0.37659.
